# The IL-1/IL-1 receptor axis and tumor cell released inflammasome adaptor ASC are key regulators of TSLP secretion by cancer associated fibroblasts in pancreatic cancer

**DOI:** 10.1186/s40425-019-0521-4

**Published:** 2019-02-13

**Authors:** Emanuela Brunetto, Lucia De Monte, Gianpaolo Balzano, Barbara Camisa, Vincenzo Laino, Michela Riba, Silvia Heltai, Marco Bianchi, Claudio Bordignon, Massimo Falconi, Attilio Bondanza, Claudio Doglioni, Maria Pia Protti

**Affiliations:** 1 Tumor Immunology Unit, Istituto di Ricovero e Cura a Carattere Scientifico (IRCCS) San Raffaele Scientific Institute, Via Olgettina 58, 20132 Milan, Italy; 2 Division of Immunology, Transplantation and Infectious Diseases, IRCCS San Raffaele Scientific Institute, Via Olgettina 58, 20132 Milan, Italy; 3 Pancreatic Surgery Unit and Pancreas Translational & Clinical Research Center, IRCCS San Raffaele Scientific Institute, Milan, Italy; 4 Division of Experimental Oncology, IRCCS San Raffaele Scientific Institute, Milan, Italy; 5 Innovative Immunotherapies Unit, IRCCS San Raffaele Scientific Institute, Milan, Italy; 6 Center for Translational Genomics and Bioinformatics, IRCCS San Raffaele Scientific Institute, Milan, Italy; 7 Chromatin Dynamics Unit, IRCCS San Raffaele Scientific Institute, Milan, Italy; 8 Division of Genetics and Cell Biology, IRCCS San Raffaele Scientific Institute, Milan, Italy; 9 MolMed SpA, Milan, Italy; 10 Pathology Unit, IRCCS San Raffaele Scientific Institute, Milan, Italy; 11 Vita-Salute San Raffaele University, Milan, Italy

**Keywords:** Pancreatic cancer, Th2 inflammation, Thymic stromal lymphopoietin, Cancer associated fibroblasts, IL-1, Inflammasome, Inflammasome adaptor ASC (apoptosis-associated speck-like protein containing a caspase-activating recruitment domain)

## Abstract

**Background:**

The thymic stromal lymphopoietin (TSLP), a key cytokine for development of Th2 immunity, is produced by cancer associated fibroblasts (CAFs) in pancreatic cancer where predominant tumor infiltrating Th2 over Th1 cells correlates with reduced patients’ survival. Which cells and molecules are mostly relevant in driving TSLP secretion by CAFs in pancreatic cancer is not defined.

**Methods:**

We performed in vitro, in vivo and ex-vivo analyses. For in vitro studies we used pancreatic cancer cell lines, primary CAFs cultures, and THP1 cells. TSLP secretion by CAFs was used as a read-out system to identify in vitro relevant tumor-derived inflammatory cytokines and molecules. For in vivo studies human pancreatic cancer cells and CAFs were orthotopically injected in immunodeficient mice. For ex-vivo studies immunohistochemistry was performed to detect ASC (apoptosis-associated speck-like protein containing a caspase recruitment domain) expression in surgical samples. Bioinformatics was applied to interrogate published data sets.

**Results:**

We show in vitro that IL-1α and IL-1β released by pancreatic cancer cells and tumor cell-conditioned macrophages are crucial for TSLP secretion by CAFs. Treatment of immunodeficient mice orthotopically injected with human IL-1 positive pancreatic cancer cells plus CAFs using the IL-1R antagonist anakinra significantly reduced TSLP expression in the tumor. Importantly, we found that pancreatic cancer cells release alarmins, among which ASC, able to induce IL-1β secretion in macrophages. The relevance of ASC was confirmed ex-vivo by its expression in both tumor cells and tumor associated macrophages in pancreatic cancer surgical samples and survival data analyses showing statistically significant inverse correlation between ASC expression and survival in pancreatic cancer patients.

**Conclusions:**

Our findings indicate that tumor released IL-1α and IL-1β and ASC are key regulators of TSLP secretion by CAFs and their targeting should ultimately dampen Th2 inflammation and improve overall survival in pancreatic cancer.

**Electronic supplementary material:**

The online version of this article (10.1186/s40425-019-0521-4) contains supplementary material, which is available to authorized users.

## Background

Pancreatic ductal adenocarcinoma (PDAC) is a very aggressive disease with a still dismal prognosis [1]. Indeed, while new chemotherapeutic regimens recently obtained significant improvements in the treatment of patients with metastatic disease [2, 3], the 5-year survival rate remains about 6%. Thus, the identification of new targetable factors and mechanisms of tumor progression is highly needed.

“Tumor-promoting inflammation” and “avoiding immune destruction” were highlighted as enabling characteristics of cancer [4]. However, inflammation varies in cellular and cytokine networks and molecular drivers in different tumors [5]. Mechanisms of “tumor-promoting inflammation” have been described in PDAC, where activation of oncogenic *Kras* promotes production of inflammatory cytokines, such as IL-6 and IL-1α, which activate STAT3 and NF-κB, respectively, in an autocrine or paracrine manner to promote tumor cell survival and proliferation, angiogenesis and increased invasive and metastatic behavior of pancreatic cancer cells [6–9].

We previously reported [10] that the ratio of Th2 over Th1 lymphoid cells in the tumor stroma is an independent predictive factor of survival after surgery in PDAC patients. Patients with a ratio superior to the median value had a statistically significant reduced survival, implying predominant Th2 inflammation as a relevant tumor-promoting factor in PDAC. Indeed, PDAC is highly infiltrated by Th2 cells and tumor associated macrophages (TAMs) of M2 type [10–13].

We found that Th2 inflammation depends on a complex crosstalk within the tumor microenvironment and tumor-draining lymph nodes [10, 14, 15] with a central role exerted by the thymic stromal lymphopoietin (TSLP) [16]. Indeed, we showed that TSLP was released by cancer associated fibroblasts (CAFs), following their activation by tumor-derived inflammatory cytokines and that, in turn, TSLP favored the conditioning of tumor infiltrating TSLP receptor-expressing dendritic cells (DCs) endowed with Th2 polarizing capability [10, 16]. These data highlighted the importance of inflammatory cytokines present in the tumor microenvironment as the first step in the development of Th2 inflammation. However, although several cytokines have been reported to regulate TSLP secretion in other models [17], which are the most relevant inflammatory cytokines, molecules and cells involved in this regulation in pancreatic cancer is not completely elucidated.

Here we show that IL-1α and IL-1β derived from tumor cells and tumor cell-conditioned macrophages is key for TSLP production by CAFs and blockade of IL-1 in vivo significantly reduced TSLP expression in the tumor. Importantly, we found that a relevant molecule driving IL-1β secretion by macrophages is the inflammasome adaptor ASC (apoptosis-associated speck-like protein containing a caspase recruitment domain), which can be released by ASC expressing pancreatic cancer cells.

## Methods

### Cells and culture media

BxPC-3, Hs766T, Capan-1, MIA PaCa-2, and THP-1 (human monocytic cell line) cell lines were purchased from the American Type Culture Collection. Paca-44, PT45, HPAF and A8184 cell lines were kindly provided by Dr. Piemonti (San Raffaele Scientific Institute). Cell lines were cultured in IMDM 10% fetal bovine serum (FBS) (Lonza) and, in the case of THP-1, β-mercaptoethanol (50 mM) (Sigma). Primary cultures of tumor cells (PCC#353 and PCC#406) and CAFs were established from tumor samples collected at surgery, as described in [10]. Briefly, tumor pieces were put in culture in IMDM medium (Lonza) plus 10% FBS and CAFs obtained by outgrowth. Alternatively, to obtain distinct cell populations after few passages tumor cells and CAFs were separated with anti-fibroblast Ab-coated beads (Miltenyi Biotec). Primary tumor cells and CAFs were characterized by western blot (WB) for expression of pan-cytokeratin and α-SMA, respectively, as shown in [10]. Cell lines were periodically tested for Mycoplasma contamination using the MycoAlert™ Mycoplasma Detection kit (Lonza).

### Real-time PCR in tumor cells

Total RNA was extracted using RNeasy Plus Mini kit (Qiagen) and 1 μg of RNA was retrotranscribed using the High-Capacity cDNA reverse transcription kit (Applied Biosystem). 50 ng cDNA were used for real-time PCR. TaqMan Fast Advanced Master mix (4,444,557, Applied Biosystem) and TaqMan primers specific for human IL-1α (Hs00174092ml), IL-1β (Hs00174097ml), TNF-α (Hs001174128ml), IL-18 (Hs01038788m1), ASC (Hs00203118ml) and GAPDH (Hs99999905m1) (Applied Biosystem) were used. Real-time PCR was performed on an AB7900HT machine (Applied Biosystem), using the SDS 2.1 software for the analysis. Target gene values were normalized with GAPDH values. Fold induction was calculated using the 2^-ΔΔCt^ method.

### siRNA transfection

SiRNA transfection of tumor cells was performed with 2000 or RNAiMax lipofectamines (Invitrogen), following manufacturer’s instructions. Briefly, 5 × 10^5^ cells/ml were cultured in 6-well plates in complete IMDM medium. 25–100 pmol IL-1α (ID: s7266), IL-1β (ID: s7269), ASC (ID: 44415) Silencer Select predesigned siRNAs or Silencer® Select Negative Control (negative siRNA) (Ambion) were used for transfection. 24 h (h) after transfection, cells were harvested and gene expression evaluated by qRT-PCR using IL-1α, IL-1β, and ASC specific TaqMan primers (Additional file 1: Figure. S1 and Additional file 2: Figure S2) or the medium replaced and cells incubated for 48-72 h. Target gene values were normalized with GAPDH values. Supernatants were collected 72 h after transfection while necrosis supernatants were obtained, as described below, after 48 h from transfection.

### Cytokine quantification in tumor cells

Cytokine production was assessed in the supernatant of viable or necrotic tumor cells and in tumor cell lysates. To obtain supernatants of viable cells, cells were plated in 6-well-plates at 8 × 10^5^ cells/well and cultured in 1,5 ml IMDM 10% FBS for 96 h. To obtain supernatant from necrotic cells, 10^6^ cell/ml of medium were treated with 3 freeze/thaw cycles and supernatant was recovered after centrifugation at 1600 rpm for 5′. To obtain cell lysates, 10^6^ cells/ml were lysed with 1 ml TritonX100 0.5% (Enzo Life Science) and clarified by centrifugation at 13.000 rpm for 20′. The following ELISA kits were used: IL-1α (DY200) and IL-1β (DY201) (R&D System), IL-18 (7620) (MBL) and TNF-α (3510-1A-20) (MabTech).

### CAF stimulation

CAFs were seeded at 1.5-3 × 10^4^ cells/well in IMDM 10% FBS in 96-well-plates and starved overnight. The next day, medium was replaced and left for 72 h with the following stimuli: recombinant human IL-1α, IL-1β and TNF-α (R&D System), IL-18 (MBL) (all in IMDM 2% FBS at the indicated concentrations), and the supernatant of necrotic PDAC cells (100 μl). In inhibition experiments, 10 μg/ml anti-IL-1α (R&D System), anti-IL-1β, anti-TNF-α or isotype-matched antibodies (Abs) (BD), and 10 μg/ml Anakinra (Kineret, Amgen Europe) were added. mRNA expression of short and long TSLP isoforms by CAFs untreated or activated by recombinant cytokines was assessed by Real Time PCR (SYBR green, Applied Biosystems) using the primers described in [18]*.* In another set of experiments, CAFs were stimulated with the supernatant of THP1 cells treated with the supernatant of tumor cells after treatment with mock-transfected or ASC siRNA or negative control siRNA. TSLP secretion in CAF supernatants was measured by ELISA (DY1398, R&D System).

### In vivo experiments

Experimental protocols were approved by the Institutional Animal Care and Use Committee. 2 × 10^6^ Hs766T (IL-1 positive) cells or 2 × 10^6^ CAFs or 2 × 10^6^ Hs766T cells plus 2 × 10^6^ CAFs were injected in the pancreas of immunodeficient NSG mice (The Jackson Laboratory). Mice implanted with Hs766T cells plus CAFs were administered anakinra daily at 10 or 25 mg/kg in the peritoneum starting 1 day post-transplantation. Mice were sacrificed after 5 days of treatment and pancreata collected. For histology, pancreata were fixed in 10% formalin followed by paraffin embedding and hematoxylin/eosin staining. For RNA extraction, inoculation sites were excised, put in RNA later and RNA was extracted using the Ribo Pure kit (Ambion). 2 μg RNA was retro-transcribed with High Capacity cDNA reverse transcription kit and 200 ng cDNA were used for Real-time PCR. TaqMan primers for human TSLP (Hs00263639m1) and HPRT1 (Hs02800695m1), mouse TSLP (Mm0049873 9 m1) and HPRT1 (Mm0044696 8 m1) (Applied Biosystem) were used. Target gene values were normalized with HPRT1 values. Fold induction was calculated using the 2^-ΔΔCt^ method.

### Western blot analysis

Protein extraction was performed using Ripa Buffer and Protease/Phosphatase Inhibitor Cocktail (Cell Signaling) and protein quantification using the Bradford method. Protein samples (30 μg) were mixed 1:1 with Laemmli buffer supplemented with 20% 1,4-Dithiothreitol (DTT) (Sigma) and loaded on 4–12% Tris-Glycine gels (Lonza). Running was performed at 125 V for 80′ and wet transfer at 25 V for 120′. Membranes were blocked with 5% milk in TBS-t for 1 h, at room temperature (RT). The primary Abs used were: anti-caspase-1 (2225, Cell Signaling), anti-ASC (AG-25B-0006, Adipogen) and anti-β-tubulin (2128, Cell Signaling). The secondary Abs were: peroxidase goat anti-rabbit IgG (H + L) (PI-1000) and peroxidase horse anti-mouse IgG (H + L) (PI-2000, Vector Laboratories). For detection we used ECL Select Reagent (Amersham, GE Healthcare), ChemiDoc™ MP system and Image Lab Software (Bio-Rad).

### Tumor cell supernatant precipitation

Tumor cell lines were plated at 2 × 10^6^ cells/well in a 6-well-plate in 2 ml IMDM 10% FBS and incubated at 37 °C for 3 *h. Medium* was replaced with 2 ml/well of Optimem+Glutamax (Gibco, Life Technologies) and cells incubated for 96 h. Supernatants were collected and trichloroacetic acid (TCA) precipitated (12% final concentration). After centrifugation at 14.000 g for 20′ at 4 °C, supernatants were aspirated and pellets washed twice with cold 80% acetone. At the end of the second wash, pellets were air dried and then dissolved in Laemmli buffer supplemented with 20% DTT (Sigma). WB analysis was performed as described above.

### Immunofluorescence

THP-1 and pancreatic cancer cells were grown in chamber slides for 24 h, fixed with 4% PAF (Electron Microscopy Sciences) for 15′ at 4 °C and washed with PBS. Permeabilization was performed in TritonX100 0,5% (30′), followed by 30′ blocking with 1% BSA (Sigma). The primary anti-ASC Ab (AG-25B-0006; Adipogen) was incubated 1 h at RT, the secondary Ab (Alexa Fluor^R^ 555 donkey anti-mouse IgG (H + L) (A31570, Invitrogen) was incubated 30′ at RT. Slides were mounted with VECTASHIELD Mounting Medium containing DAPI (Vector Laboratories). Images were acquired using Axio Observer Z.1 Microscope (Zeiss) and Volocity Software (PerkinElmer). THP1 cells were stimulated with 1 μg/ml LPS for 24 h.

Deparaffinated histologic sections after antigen retrieval were incubated for 1 h at RT with the primary anti-ASC polyclonal Ab (ADI-905-173-100; Enzo) or IL-1beta polyclonal Ab (SC 7884 H153 SantaCruz) and anti-CD163 mouse monoclonal Ab (clone 10D6 cod NCL-L-CD163; Leica Biosysistems) followed, after rinsing in PBS, by the secondary Abs (Alexa Fluor^R^ 555 donkey anti-mouse IgG, cod. A31570 and Alexa Fluor 488 goat anti-rabbit IgG cod A^− 11,034^, Invitrogen) for 30′ and mounted as above. Images were captured with a fluorescence-equipped i80 Eclipse Nikon instrument.

### Macrophage stimulation

THP-1 cells were plated at 10^5^ cells/well in 96-well-plates in 100 μl IMDM 10% FBS without/with 50 nM PMA (phorbol 12-myristate 13-acetate, Sigma) and incubated at 37 °C for 18 h. Cells were then incubated for additional 24 h with the following stimuli: 1 μg/ml LPS and 96 h supernatant from PDAC cell lines (100 μl). In inhibition experiments, anti-IL-1α (10 μg/ml), anti-TNF-α (10 μg/ml), anti-IL-18 (10 μg/ml) and BoxA (10 ng/ml) or iso-Ig Abs (10 μg/ml) were added directly in the culture or THP1 cells were stimulated with ASC- or irrelevant iso-Ig Ab-depleted supernatants (100 μl). Depleted supernatants were obtained as follows: cell-free BxPC-3 and A8184 supernatants (from 72 h culture in IMDM 10% FBS) were incubated for 2 h at RT in 96-well-plates (not-treated sterile Costar #3788) coated with 1 μg/ml anti-ASC Ab (AL177 Adipogen) or normal rabbit control IgG (Peprotech), supernatants were then collected and stored at − 20 °C. THP1 were also stimulated with the supernatant of tumor cells that had been previously treated with either ASC siRNA or a negative control siRNA. IL-1β in the supernatants of THP1 cells was measured by ELISA.

### Immunohistochemistry

Tissue microarrays were produced from paraffin-embedded tissue blocks from 41 PDAC surgical specimens. All cases were immunostained with a sensitive non-biotin detection system (NovoLink polymer, Novocastra), with diaminobenzidine development. Heat induced antigen retrieval was performed using citrate buffer 0.01 M, pH 6.0 or Tris-EDTA pH 9.0 in a water bath for 30′. Immunostaining was performed using the anti-ASC Ab (Enzo Life Science) and Ultravision Quanto Detection System HRP polymer (Thermo Scientific). Samples were considered positive if the percentage of positive cells was superior to 10%.

### Statistical analysis and bioinformatics

Statistical analyses were performed using Mann Whitney test, Wilcoxon matched-pairs signed rank test and paired or unpaired Student’s *t*-test, as specified in the figure legends. Data regarding RNA sequencing of 178 PDAC primary samples have been taken from the TCGA Research Network (http://cancergenome.nih.gov/) through R statistical programming environment (https://www.R-project.org) using TCGA2STAT package. A subset of the “Clinical Data Table”, containing only information about overall survival has been used. The survival curves were estimated according to the Kaplan-Meier method and compared using the log-rank test. Statistical analyses were performed using GraphPad Prism version 5.0 for Mac (GraphPad Software). Values of *p* < 0.05 were considered significant.

## Results

### TSLP secretion by CAFs induced by tumor cell supernatant mainly depends on IL-1α

Two TSLP isoforms (short and long) have been described in humans [19, 20]. The short isoform is mainly expressed in steady state conditions mediating homeostatic functions, whereas the long isoform is upregulated in inflammatory conditions [19, 20]. Due to the different physiologic and pathologic functions exerted by the two TSLP isoforms, we investigated at the transcription level their expression in CAFs in steady state and activated conditions (Additional file 3: Figure S3). We found that expression of the short form was detectable at variable levels, depending on the CAF, at the steady state and did not significantly increase after activation (Additional file 3: Figure S3a). The expression of the long form was low at the steady state and highly and significantly increased after activation (Additional file 3: Figure S3a). When we compared the fold increase calculated from the activated and basal levels of expression, we found a significant difference between the long and short TSLP isoforms (Additional file 3: Figure S3b). These results, along with the notion that at the steady state we found negligible TSLP secretion by CAFs, support a predominant role for the inducible long isoform of TSLP in pancreatic cancer where inflammation takes place. However, we cannot exclude that in certain conditions the short TSLP isoform might exert homeostatic and anti-inflammatory functions worthwhile further investigations.

**Fig. 1:**
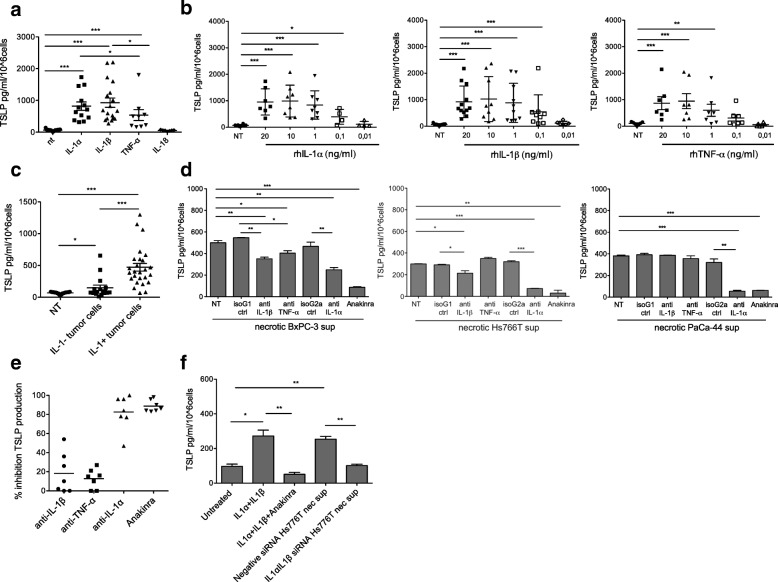
Tumor cell-derived IL-1 has a key role in TSLP secretion by CAFs. **a** TSLP secretion by CAFs (*n* = 18) either untreated (NT) or treated with the indicated cytokines (20 ng/ml). Each symbol refers to a single experiment and represents the mean of duplicate determinations. Significance was determined using Mann Whitney test. **b** TSLP secretion by CAFs in the absence (NT) or in the presence of increasing doses of recombinant IL-1α, IL-1β and TNF-α. Significance was determined using Mann Whitney test. **c** TSLP secretion by CAFs (*n* = 14) either untreated (NT) or treated with supernatants of necrotic IL-1 negative (IL-1-) (*n* = 16) or positive (IL-1+) (*n* = 26) tumor cells. Each symbol refers to a single experiment and represents the mean of duplicate determinations. Significance was determined using Mann Whitney test. **d** TSLP secretion by CAFs induced by necrotic tumor cell-derived supernatants in the absence (NT) or in the presence of the indicated neutralizing Abs or isotype control Abs or the IL-1RA (Anakinra). Representative experiments performed with the indicated tumor cell lines. Error bars represent standard deviations of triplicate values. Significance was determined using Student’s *t*-test. **e** Cumulative results: each dot refers to a single experiment and represents the percentage of inhibition calculated on the value of TSLP secretion in the presence of the isotype control antibody. **f** TSLP secretion by CAFs either untreated or stimulated with the supernatant of necrotic Hs766T cells after treatment with IL-1α/IL-1β siRNA or negative control siRNA. Treatment of CAFs with recombinant IL-1α + IL-1β (20 ng/ml each) in the absence or in the presence of anakinra were used as controls. Error bars represent standard deviations of triplicate values. Significance was determined using Student’s *t*-test. Values significantly different were indicated as: *p < 0.05, **p < 0.01 and ***p < 0.001

To evaluate the role of inflammatory cytokines in inducing TSLP secretion by CAFs we first measured the mRNA expression and protein secretion of IL-1α, IL-1β, IL-18 and TNF-α from ten PDAC cell lines comprising two established from primary tumors. IL-1α and/or IL-1β were expressed in 6 out of 10 lines, IL-18 in 9 and low levels TNF-α in 6 (Additional file 4: Figure S4a). Cytokine secretion in cell lysates (Additional file 4: Figure S4b) mostly paralleled the mRNA expression data, whereas the amount of cytokines released in the supernatants, with the exception of IL-1α, was very low or undetectable (Additional file 4: Figure S4c). We then stimulated CAFs with high dose recombinant cytokines and found that, although at different extent, IL-1α, IL-1β and TNF-α induced TSLP secretion while IL-18 did not (Fig. 1a). As the level of cytokines released by PDAC cells is relatively low (Additional file 4: Figure S4b-4c), we performed dose response curves and found that TSLP secretion was still detectable at concentrations usually present in tumor cell lysates and as low as 0.1 ng/ml for IL-1α and IL-1β, while TNF-α showed a statistically significant effect only down to 1 ng/ml (Fig. 1b). Next, as necrosis is frequently present in PDAC due to the highly hypoxic tumor microenvironment [21], we stimulated CAFs with the supernatant of necrotic PDAC cells (i.e., BxPC3, Hs766T, PaCa-44, PT45, MIA PaCa-2, HPAF and A8184), where the levels of cytokines were comparable or even higher to those measured in cell lysates (not shown and Additional file 4: Fig. 4b). Supernatants from necrotic cells, independently of IL-1 release, induced significant TSLP secretion by CAFs. However, the supernatants from IL-1-secreting cells (i.e., BxPC3, Hs766T and PaCa-44) were more effective (Fig. 1c). To investigate the relevance of each cytokine anti-IL-1α, anti-IL-1β, anti-TNF-α Abs and the recombinant form of the IL-1 receptor antagonist (IL-1RA) anakinra were added in culture to inhibit TSLP secretion by CAFs activated with the supernatant of BxPC3, Hs766T and Paca-44 cells, which release the highest amounts of IL-1α, IL-1β and TNF-α (Additional file 4: Figure S4b). We found that TSLP secretion induced by the supernatant of BxPC-3 was slightly, although significantly, inhibited by anti-IL1β and anti-TNFα Abs and strongly inhibited by anti-IL1α Ab and anakinra (Fig. 1d, left), TSLP secretion induced by the supernatant of Hs766T was slightly inhibited by anti-IL-1β Ab and very strongly by anti-IL1α Ab and anakinra (Fig. 1d, middle), and TSLP secretion induced by the supernatant of PaCa44 was strongly and equally inhibited by anti-IL1α Ab and anakinra (Fig. 1d, right). Independent experiments repeated with the 3 cell lines and different CAFs showed that the highest percentage of TSLP inhibition was obtained with the anti-IL-1α Ab and anakinra (Fig. 1e), suggesting that the IL-1/IL-1R axis is key for TSLP secretion by CAFs. This finding was further confirmed by experiments in which TSLP secretion by CAFs was induced by the supernatant of tumor cells after silencing of IL-1α and IL-1β expression by siRNA. In agreement with the inhibition experiments using anti-IL-1 Abs and anakinra (Fig. 1d, middle), TSLP secretion in the presence of the supernatant of IL-1α/IL-1β silenced Hs776T cells was completely abolished (Fig. 1f).

**Fig. 2:**
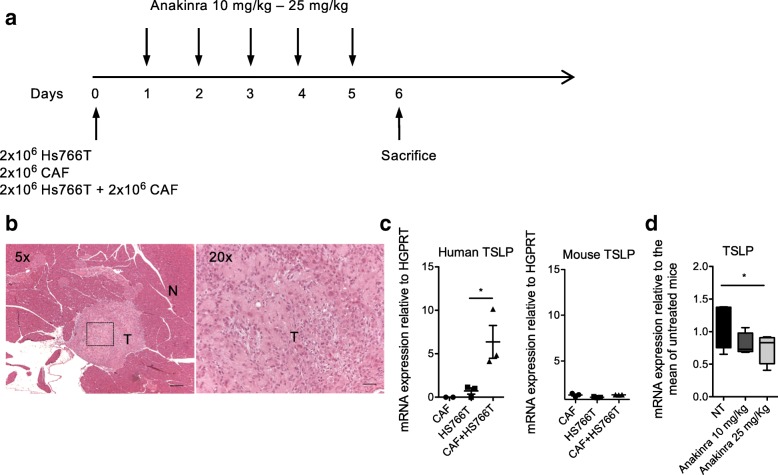
Treatment of immunodeficient NSG mice orthotopically transplanted with IL-1 positive Hs766T cells plus CAFs with anakinra reduces TSLP expression in vivo. **a** Treatment schedule. Mice were orthotopically injected on day 0 with Hs766T cells plus CAFs. Hs766T cells alone and CAFs alone were injected as controls. The next day mice transplanted with Hs766T cells plus CAFs started intraperitoneal injection with the two indicated doses of anakinra and mice were sacrificed on day 6 after daily administration. **b** Hematoxylin and eosin staining of the pancreas. Left, a tumor mass (T) localized within the normal pancreas (N) is evident. Right, at higher magnification, histological features of pancreatic cancer with cancer cells, fibroblasts and extracellular matrix can be recognized. **c** Human (left) and mouse (right) TSLP mRNA expression in pancreata of NSG mice implanted with CAFs alone (*n* = 3), Hs766T cells alone (*n* = 3) or CAFs plus Hs766T cells (*n* = 3). Significance was determined using Student’s *t*-test. **d** TSLP expression in pancreata of NSG mice untreated (NT) (*n* = 4) or treated with the two indicated doses of Anakinra (*n* = 4 for each dose). Significance was determined using Student’s *t*-test. Values significantly different were indicated as: *p < 0.05

We then evaluated the relevance of tumor-derived IL-1 in driving TSLP secretion by CAFs in vivo by using immunodeficient NSG mice orthotopically injected with IL-1 positive human PDAC cells plus CAFs and treated with anakinra, following the scheme depicted in Fig. 2a. As PDAC cells we chose the Hs766T, based on the previous experiments (Fig. 1d-f), and because, when orthotopically injected in immunodeficient mice, show 90% intrapancreatic tumorigenicity [22]. Primary CAFs were co-injected with Hs766T cells. On day 6 mice were sacrificed and the tumor excised. Hematoxylin and eosin staining confirmed the presence of tumor masses within normal murine pancreatic parenchyma composed by tumor cells, CAFs and extracellular matrix (Fig. 2b). We first verified that interaction between Hs766T cells and CAFs induced TSLP up-regulation in vivo. Indeed, human TSLP mRNA expression in lesions developed after co-injection of Hs766T cells and CAFs was significantly higher compared with those from mice injected with Hs766T cells or CAFs alone (Fig. 2c, left). Murine TSLP mRNA expression evaluated in the same samples did not show significant differences, suggesting that murine cells were not induced to express TSLP in our system (Fig. 2c, right). We then evaluated the effect of the treatment with anakinra on TSLP expression in mice co-injected with Hs766T cells and CAFs and we found that tumors from mice treated with anakinra expressed a significantly lower amount of human TSLP compared to untreated mice (Fig. 2d), confirming in vivo the key role of the IL-1/IL-1R axis in TSLP secretion.

### Tumor cells release alarmins, among which ASC, for inflammasome activation and IL-1β secretion by macrophages

As not all tumor cells constitutively express/release IL-1 (Additional file 4: Figure S4), we hypothesized that tumor infiltrating myeloid cells, which are highly represented within the tumor microenvironment [13], could be an alternative source of IL-1 for induction of TSLP secretion by CAFs. In support of this hypothesis, we previously reported mRNA IL-1β expression in both the pancreatic tumor epithelial and stromal compartments [10], and IL-1β staining within the stroma mainly in association with CD163 positive TAMs (Additional file 5: Figure S5).

Production and release of pro-inflammatory cytokines depends on NF-κB and, in the case of IL-1β, inflammasome activation [23, 24]. The inflammasome is a large multiprotein complex, which is responsible for IL-1β processing [24]. Production and release of IL-1β requires two distinct signals: the first derived from exogenous or endogenous stimuli leads to NF-κB activation and synthesis of pro-IL-1β and inflammasome components. The second signal (mediated by molecules such as ATP, uric acid and reactive oxygen species) activates the inflammasome with assembly and activation of nucleotide-binding oligomerization domain (NOD)-like receptors (NLR) family members that oligomerize interacting with the adaptor ASC that in turn recruits procaspase-1. Procaspase-1 clustering leads to the formation of the active form of caspase-1, which then processes the IL-1β pro-form to generate the biologically active protein.

**Fig. 3:**
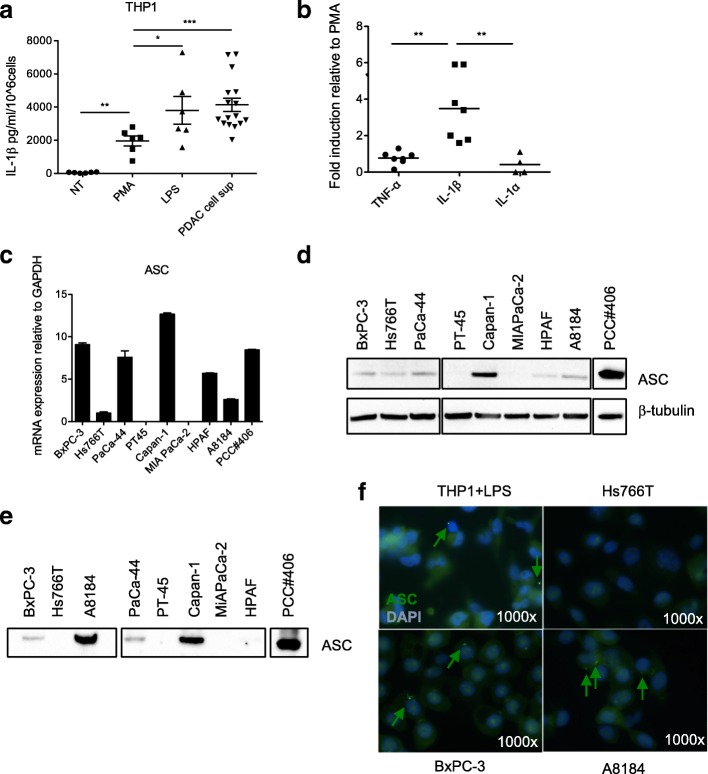
Tumor cells induce IL-1β secretion in macrophages through the release of alarmins, among which ASC. **a** IL-1β secretion by THP-1 cells after priming with PMA and stimulation with tumor cell-derived supernatants (PDAC cell sup) (*n* = 16). LPS was used as positive control. Each symbol refers to a single experiment and represents the mean of duplicate determinations. Significance was determined using Mann Whitney test. **b** Comparison of cytokine secretion by THP1 treated with PDAC cell sup, as in **a**. Each symbol refers to a single experiment and represent the mean of duplicate determinations. The values are reported as fold increase relative to those of THP1 stimulated in the presence of PMA only. Significance was determined using Mann Whitney test. **c** ASC mRNA expression in the indicated PDAC cell lines. **d** WB analysis of ASC protein in the indicated PDAC cell lines. **e** WB analysis to determine levels of ASC protein in TCA precipitated supernatants of the indicated PDAC cell lines. **f** Immunofluorescence analysis of ASC expression in LPS treated THP-1 (positive control) and the indicated PDAC cell lines. ASC aggregates/specks are represented by green dots (arrows)

We asked which signals were responsible for IL-1β production and secretion by TAMs. To this aim we performed in vitro studies and took advantage of the human monocytic cell line THP1, as a model cell. THP1 were first primed with PMA and then activated with LPS, as a positive control, or the supernatant of viable tumor cell lines (i.e., BxPC-3, Hs766T, PaCa-44, MIA PaCa-2, A8184, PCC#353 and PCC#406). The supernatants of tumor cells induced the secretion of high levels of IL-1β by THP1 cells (Fig. 3a). When we checked also for secretion of IL-1α and TNF-α we found that IL-1β release was significantly higher compared to the other inflammatory cytokines (Fig. 3b). Collectively, these data suggest that tumor cells secrete/release alarmins which predominantly induce IL-1β secretion by macrophages.

To understand which molecules were responsible for this activity we performed experiments of THP1 activation with tumor cell supernatants in the presence of neutralizing Abs and BoxA to inhibit IL-1α, TNF-α, IL-18 and HMGB1, respectively, which are released by tumor cells (Additional file 4: Figure S4 and Additional file 6: Figure S6). We did not detect any inhibitory effect (data not shown), and this was in agreement with the lack of IL-1β secretion by THP1 cells when stimulated with recombinant IL-1α, TNF-α, IL-18 and HMGB1 (data not shown).

It has been recently reported that ASC can assemble and form specks that are released by dying cells to reinforce the inflammatory cascade [25, 26], and phagocytosis of ASC specks by macrophages led to IL-1 maturation through caspase-1 activation [25, 26]. We evaluated the ASC mRNA in PDAC cell lines and found expression in 7 out of 9 tested (Fig. 3c) that was confirmed at the protein level in tumor cell lysates (Fig. 3d). To verify whether ASC in PDAC cells forms aggregates that can be released, we assessed the presence of ASC protein in the supernatant of viable tumor cells. We detected ASC release from 5 of the 7 ASC positive tumor cell lines (Fig. 3e). The formation of ASC specks in PDAC cell lines with features similar to those observed in LPS stimulated THP1 cells, as shown in [25, 26], was confirmed by immunofluorescence. In agreement with the previous results, we observed ASC specks in tumor cell lines releasing low (BxPC-3) and high (A8184) amounts of ASC in the supernatant but not in Hs766T that did not release ASC (Fig. 3f). Collectively, these data suggest that tumor cell-derived ASC can be released in the extracellular space in a high percentage of ASC positive tumor cell lines.

**Fig. 4:**
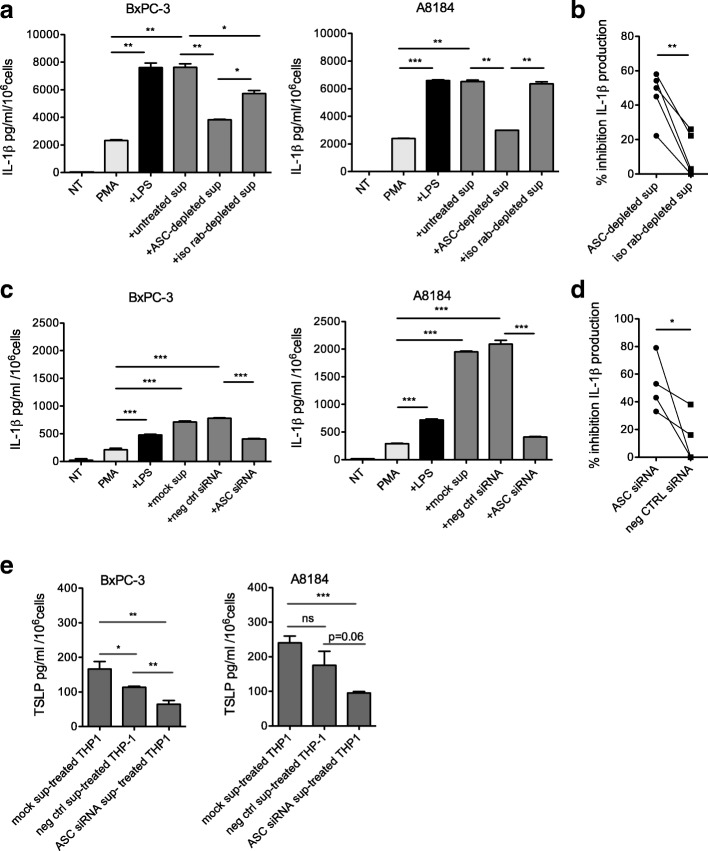
Tumor cell-derived ASC induces IL-1β secretion by macrophages and indirectly TSLP secretion by CAFs. **a** IL-1β secretion by THP-1 cells in the presence of the indicated stimuli, i.e. none (NT), PMA alone, PMA + LPS, PMA+ tumor cell sup (untreated sup), ASC-depleted or iso-rab-depleted tumor cell sups. The experiments were performed with BxPC3 (left) and A8184 (right) cell lines. Error bars represent standard deviations of triplicate values. Significance was determined using Students’ *t* test. **b** Cumulative results of independent experiments (each symbol refers to a single experiment). Percentage of inhibition was calculated on the values of THP1 in the presence of untreated sup. Significance was determined using paired Students’ *t* test. **c** IL-1β secretion by THP-1 cells in the presence of the indicated stimuli, i.e. none (NT), PMA alone, PMA + LPS, the supernatant (sup) of tumor cells (BxPC3 and A8184) either mock transfected (mock sup) or transfected with negative control siRNA (neg ctrl siRNA) or ASC siRNA. Error bars represent standard deviations of triplicate values. Significance was determined using Students’ *t* test. **d** Cumulative results of independent experiments (each symbol refers to a single experiment). Percentage of inhibition was calculated on the values of THP1 in the presence of mock-sup. Significance was determined using paired Students’ *t* test. **e** TSLP secretion by CAFs stimulated with the supernatant of THP1 cells treated with the supernatant of the indicated tumor cell lines (BxPC-3 and A8184) either mock transfected (mock sup) or transfected with control siRNA (neg ctrl sup) or ASC siRNA sup (*n* = 3). Error bars represent standard deviations of triplicate values. Significance was determined using unpaired Students’ *t* test. Values significantly different were indicated as: *p < 0.05, **p < 0.01 and ***p < 0.001

To investigate whether tumor cell-released ASC could act as alarmin for IL-1β secretion by THP-1 we stimulated the cells with untreated or ASC-depleted or isotype control Ab-depleted viable tumor cell supernatants. We found that ASC-depleted compared to untreated or isotype control Ab-treated supernatants induced a significant reduced level of IL-1β secretion (Fig. 4a-b), demonstrating that ASC has a role in ASC- releasing PDAC cell supernatant-depended IL-1β secretion by THP1. These results were confirmed when THP1 cells were stimulated with the supernatant of ASC-silenced tumor cells (Fig. 4c-d), reaching levels of inhibition of IL-1β secretion similar to those obtained with the depleting Abs (Fig. 4b-d). Finally, we assessed the relevance of tumor cell-derived ASC in the induction of TSLP secretion by CAFs. We found that TSLP secretion was significantly reduced when CAFs were activated with the supernatants of THP1 after treatment with the supernatant of ASC-silenced tumor cells (Fig. 4e), suggesting that indeed ASC expression and release by tumor cells indirectly contributed to TSLP secretion by CAFs.

**Fig. 5:**
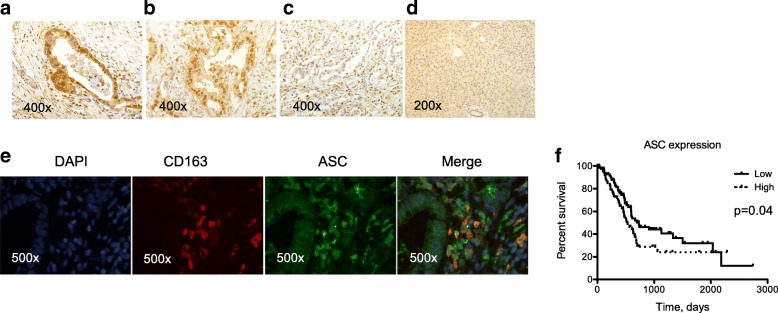
ASC is expressed in situ in tumor cells and TAMs and its expression in primary tumors correlates with reduced overall survival. **a**-**d** Immunohistochemistry of ASC protein expression in representative samples of tumor and tumor surrounding tissue. **a** ASC staining in both the nucleus and the cytoplasm in both tumor cells and TAMs. **b** ASC staining in both the nucleus and the cytoplasm in tumor cells while prevalently cytoplasmic in TAMs. **c** ASC staining is negative in tumor cells and in TAMs is prevalently nuclear. **d** ASC staining in normal pancreatic tissue surrounding the tumor. **e** Immunofluorescence staining of ASC (green) in tumor cells and TAMs (identified by CD163 staining in red). Cells co-expressing ASC and CD163 are indicated in orange (merge). **f** Kaplan-Meier curves of overall survival of 178 PDAC patients grouped based on median ASC RNA expression. Significance was evaluated by the Log-rank (Mantel-Cox) test (*p* = 0.04)

Lastly, we performed immunohistochemistry for ASC expression in 41 surgical specimens from primary tumors collected in our hospital (Fig. 5a-d). We detected ASC staining in tumor cells as well as in TAMs (identified by CD163 staining, Fig. 5e) within the stroma. In tumor cells ASC was detected in a high percentage of tumor samples (about 90%) in both the nucleus and the cytoplasm (Fig. 5a-b). In TAMs ASC staining was always detectable and expressed in both the nucleus and the cytoplasm (Fig. 5a) or prevalently in the cytoplasm (Fig. 5b) or in nucleus (Fig. 5c). Normal pancreatic tissue surrounding the tumor (i.e., macroscopically uninvolved) showed a weak diffuse staining (Fig. 5d). To evaluate the clinical relevance of ASC expression in vivo we performed bioinformatics and survival analysis based on data of gene expression (RNA sequencing) of 178 PDAC samples available from the TCGA Research Network. As shown in Fig. 5f, when patients were stratified in low and high ASC mRNA expression based on median values, patients with low expression had statistically significant increased overall survival.

Collectively, the data suggest that extracellular ASC released by tumor cells induces IL-1β secretion in TAMs that in turn activates TSLP by CAFs and that ASC is expressed in a high percentage of primary tumors where its expression correlates with reduced survival in PDAC patients.

## Discussion

Tumor infiltration by Th2 cells has been associated with worse prognosis in neoplastic patients bearing several tumors with the exception of Hodgkin’s lymphoma [27]. We previously reported that in PDAC predominant Th2 cells correlated with TSLP expression by CAFs and tumor infiltration by TSLP receptor-expressing DCs, pointing to TSLP as a master regulator of pro-tumor Th2 inflammation in PDAC. Recently, a study using a non-orthotopic transplantable PDAC mouse model with high levels of systemic TSLP reported impaired pancreatic cancer development associated with tumor infiltration by Th2 cells [28]. The opposing anti-tumor roles of TSLP and Th2 cells of this study compared to those we observed in human PDAC may be explained by early-stage disease and the high systemic TSLP levels that otherwise we did not find increased in PDAC patients’ sera (unpublished data).

In the present study, we report that tumor-derived IL-1α and IL-1β exert a primary role in driving TSLP secretion by CAF and that in vivo treatment with anakinra significantly reduces TSLP expression in the tumor. In pancreatic cancer IL-1 secretion by tumor cells has been shown to constitutively activate NF-κB and to increase expression of downstream genes involved in the metastatic cascade [29, 30] and chemoresistance [31]. Recently, in vivo treatment with anakinra using a PDAC orthotopic nude mouse model reduced tumor growth by inhibiting IL-1α induced NF-κB activity [32]. Here we show that, using a similar mouse model, treatment with anakinra was able to significantly reduce the expression of TSLP, establishing an additional rational for the use of anakinra in the treatment of PDAC patients. Clinical trials assessing the safety and improved survival of anakinra in combination with standard chemotherapy are ongoing (NCT02021422 and NCT02550327): whether TSLP expression and Th2 inflammation will be affected when using this drug in the neoadjuvant setting would be an interesting end point.

As a major finding of our study, we found that IL-1β availability in the tumor is significantly sustained by tumor cell-released ASC that acts as alarmin to induce and/or amplify inflammatory responses through macrophage activation. ASC was originally discovered because inducing apoptosis when overexpressed in HL-60 human leukemia cells [33] and its expression was found silenced in many human tumors by methylation of CpG island in the ASC gene [34–38], thus preventing tumor cells from undergoing apoptosis and acting as tumor suppressor. In agreement with a tumor suppressor function, ASC methylation correlated with poor prognosis in non-small-cell lung cancer [39], oral squamous cell carcinoma [40] and gastric cancer [41]. On the other hand, in another study [42], high expression of ASC in oral cavity squamous cell carcinoma correlated with poor patients’ survival and in a medulloblastoma mouse model ASC genetic deletion markedly reduced proliferation, hyperplasia and mortality [43]. In addition, in myeloid cells ASC, as an inflammasome adaptor molecule, is required for IL-1β processing and activation. Thus, in the context of cancer development, where inflammation is present, ASC may exert multiple and opposing functions as shown in epithelial skin carcinogenesis [44], being tumorigenic when expressed in infiltrating myeloid cells but tumor suppressor when expressed in keratinocytes. Even in the context of its inflammatory role, ASC may exert opposing functions in primary versus metastatic melanoma by different modulation of NF-kB activity [45]. Whether ASC expression in pancreatic cancer cells have a tumor suppressor function is not known. Interestingly, we found that ASC is expressed in a high percentage of pancreatic cancer samples in both tumor cells and TAMs and its expression correlates with reduced patients’ survival. This would suggest that overall in pancreatic cancer the predominant function of ASC is pro-inflammatory.

It has been reported that ASC specks are released into the extracellular space secondary to caspase-1-dependent cell death and that ASC specks taken up by neighboring macrophages via phagocytosis lead to the release of IL-1β, suggesting that extracellular ASC specks serve as an inflammation-initiating danger signal [25, 26]. Which is the mechanism of ASC release from PDAC cells and whether ASC in tumor cell supernatants is sufficient per se to activate both pro-IL-1β production and processing will need further investigation. It has been reported that ASC regulates NF-κB activity to either enhancement or inhibition depending on the ratio of its levels to other ASC-binding proteins [46–48]. Thus, in our system, tumor cell-derived ASC might also contribute directly through unknown mechanisms to NF-κB activation for pro-IL-1β production. We do not exclude that PDAC cells release additional alarmins/molecules helping to drive IL-1β production and inflammasome activation in macrophages, as in the case of methylated ASC pancreatic cancer cells. However, in pancreatic cancers in which ASC is expressed, this molecule appears to have a dominant role.

TSLP targeting with an anti-TSLP human monoclonal antibody has been utilized in the treatment of uncontrolled asthma with clinical improvements [49] and is currently under investigation in several studies to evaluate efficacy and safety [20]. However, due to the different functions exerted by the two TSLP isoforms, in pancreatic cancer where both isoforms can be expressed, targeting cytokines and molecules implicated in the induction of the inflammatory long TSLP isoform would be preferable. In this study we identified IL-1 derived by tumor cells and TAMs as relevant inducers of TSLP release by CAFs (Additional file 7: Figure S7, in red). Pharmacological approaches targeting the described pathways are available or under development (Additional file 7: Figure S7, in red). In addition to anakinra, which targets the IL-1R and for which clinical studies are ongoing (see above), other inhibitors targeting monocytes recruitment into the tumor are available. The CCR2 inhibitor PF-04136309 has been recently used in combination with chemotherapy to treat patients with borderline resectable or locally advanced disease (NCT01413022), demonstrating that CCR2-targeted therapy is safe and tolerable [50]. The possibility of interfering with inflammasome components, such as NLRP3, is also being pursued and recently a diarylsulfonylurea-containing compound (MCC950) was identified in preclinical studies as a specific inhibitor of the NLRP3 inflammasome and of NLRP3-dependent ASC speck formation [51]. Remarkably, NLRP3 expression by TAMs in PDAC murine models has been recently shown to exert tumor-promoting functions and its ablation significantly prolonged mice survival [52].

## Conclusions

In summary, we identified tumor-derived IL-1α and IL-1β as key cytokines in driving TSLP secretion by CAFs and tumor-released ASC, whose expression in PDAC correlates with reduced patients’ survival, as a relevant component in the inflammatory cascade involving activation of IL-1β secretion by TAMs. Therapeutic strategies interfering with these pathways are currently tested or under development and our results support a strong additional rational for their use in combination with standard of care.

## Additional files

Additional file 1: **Figure S1.** Validation of knockdown of IL-1α and IL-1β expression in Hs766T cells after siRNA transfections. Cells transfected with IL-1α + IL-1β siRNA showed significant reduction in both IL-1α and IL-1β mRNA expression. Significance was determined using Student’s *t* test. Values significantly different were indicated as: *p < 0.05 and **p < 0.01. (DOCX 192 kb)

Additional file 2: **Figure S2.** Validation of knockdown of ASC/PYCARD expression in BxPC-3 and A8184 cells after siRNA transfections. Cells transfected with ASC siRNA showed significant reduction in both ASC mRNA expression. Significance was determined using Student’s *t* test. Values significantly different were indicated as: *p < 0.05 and **p < 0.01. (DOCX 162 kb)

Additional file 3: **Figure S3.** TSLP mRNA isoform expression in CAFs in steady state conditions or after activation. For activation CAFs (*n* = 5) were treated with recombinant cytokines (IL-1α, IL-1β and TNF-α), which were used either alone or in combinations at the concentration of 20 ng/ml/each cytokine. **a** Expression of short and long TSLP forms by CAF untreated (basal) or activated by cytokine treatment (activated). **b** Fold (DOCX 191 kb) increase in TSLP mRNA expression after activation for the long and short TSLP isoforms. Significance was determined by the Wilcoxon matched-pairs signed rank test (a) and Mann Whitney test (b). Values significantly different were indicated as: *p* < 0.05. (DOCX 395 kb)

Additional file 4: **Figure S4.** Inflammatory cytokine expression and secretion by PDAC cell lines. PDAC cell lines either commercially available or established from primary surgical samples were tested for mRNA expression of IL-1α, IL-1β, IL-18 and TNF-α by real time PCR (**a**), and protein expression in lysates (**b**) or in the supernatants (**c**) detected by ELISA. (DOCX 395 kb)

Additional file 5: **Figure S5.** IL-1β expression in the stroma is mainly supported by TAMs. Immunofluorescence staining was performed to identify IL-1β secreting cells in the tumor stroma. TAM are stained in red (i.e., CD163), IL-1β in green. (DOCX 248 kb)

Additional file 6: **Figure S6.** Levels of HMGB1 measured by ELISA in the supernatant of the indicated PDAC cell lines. (DOCX 86 kb)

Additional file 7: **Figure S7.** Proposed model of crosstalk within the tumor microenvironment leading to TSLP secretion by CAFs and Th2 inflammation. **a** Inflammatory cytokines are released from dying/dead tumor cells. **b** Tumor cells release ASC that activates TAMs to secrete IL-1β (**c**). **d** Tumor cell- and TAM-derived IL-1α and IL-1β, respectively, induce TSLP secretion by CAFs. **e** TSLP activates resident DCs with Th2 polarizing capability. Pharmacological molecules able to interfere with these mechanisms are indicated in red. (DOCX 2538 kb)

## Abbreviations

ASC: Apoptosis-associated speck-like protein containing a caspase recruitment domain
CAF: Cancer associated fibroblast
h: Hour
PDAC: Pancreatic ductal adenocarcinoma
RT: Room temperature
TAM: Tumor associated macrophages
TCA: Trichloroacetic acid
TSLP: Thymic stromal lymphopoietin
WB: Western blot
