# Supplementary material for: The IL-1/IL-1 receptor axis and tumor cell released inflammasome adaptor ASC are key regulators of TSLP secretion by cancer associated fibroblasts in pancreatic cancer
Source: J Immunother Cancer. 2019 Feb 13;7:45. doi: 10.1186/s40425-019-0521-4 (PMC6373075; doi:10.1186/s40425-019-0521-4)
Supplement: Supplementary file 2 — Figure S2. Validation of knockdown of ASC/PYCARD expression in BxPC-3 and A8184 cells after siRNA transfections. Cells transfected with ASC siRNA showed significant reduction in both ASC mRNA expression. Significance was determined using Student’s t test. Values significantly different were indicated as: *p < 0.05 and **p < 0.01. (DOCX 162 kb) [file 40425_2019_521_MOESM2_ESM.docx]

**Additional file 2: Supplementary Figure S2**

**Figure S2**. Validation of knockdown of ASC/PYCARD expression in BxPC-3 and A8184 cells after siRNA transfections. Cells transfected with ASC siRNA showed significant reduction in both ASC mRNA expression. Significance was determined using Student’s *t* test. Values significantly different were indicated as: *p<0.05 and **p<0.01.
