# Supplementary material for: The IL-1/IL-1 receptor axis and tumor cell released inflammasome adaptor ASC are key regulators of TSLP secretion by cancer associated fibroblasts in pancreatic cancer
Source: J Immunother Cancer. 2019 Feb 13;7:45. doi: 10.1186/s40425-019-0521-4 (PMC6373075; doi:10.1186/s40425-019-0521-4)
Supplement: Supplementary file 3 — Figure S3. TSLP mRNA isoform expression in CAFs in steady state conditions or after activation. For activation CAFs (n = 5) were treated with recombinant cytokines (IL-1α, IL-1β and TNF-α), which were used either alone or in combinations at the concentration of 20 ng/ml/each cytokine. a Expression of short and long TSLP forms by CAF untreated (basal) or activated by cytokine treatment (activated). b Fold (DOCX 191 kb) increase in TSLP mRNA expression after activation for the long and short TSLP isoforms. Significance was determined by the Wilcoxon matched-pairs signed rank test (a) and Mann Whitney test (b). Values significantly different were indicated as: p < 0.05. (DOCX 395 kb) [file 40425_2019_521_MOESM3_ESM.docx]

**Additional file 3: Supplementary Figure S3**

a

b

**Figure S3**. TSLP mRNA isoform expression in CAFs in steady state conditions or after activation. For

activation CAFs (n=5) were treated with recombinant cytokines (IL-1β, IL-1α and TNF-α), which were used either alone or in combinations at the concentration of 20 ng/ml/each cytokine. **a** Expression of short and long TSLP forms by CAF untreated (basal) or activated by cytokine treatment (activated). **b** Fold increase in TSLP mRNA expression after activation for the long and short TSLP isoforms. Significance was determined by the Wilcoxon matched-pairs signed rank test (a) and Mann Whitney test (b). Values significantly different were indicated as: p<0.05.
