# Supplementary material for: The IL-1/IL-1 receptor axis and tumor cell released inflammasome adaptor ASC are key regulators of TSLP secretion by cancer associated fibroblasts in pancreatic cancer
Source: J Immunother Cancer. 2019 Feb 13;7:45. doi: 10.1186/s40425-019-0521-4 (PMC6373075; doi:10.1186/s40425-019-0521-4)
Supplement: Supplementary file 4 — Figure S4. Inflammatory cytokine expression and secretion by PDAC cell lines. PDAC cell lines either commercially available or established from primary surgical samples were tested for mRNA expression of IL-1α, IL-1β, IL-18 and TNF-α by real time PCR (a), and protein expression in lysates (b) or in the supernatants (c) detected by ELISA. (DOCX 395 kb) [file 40425_2019_521_MOESM4_ESM.docx]

**Additional file 4: Supplementary Figure S4**

a

b

c

**Figure S4.** Inflammatory cytokine expression and secretion by PDAC cell lines. PDAC cell lines either

commercially available or established from primary surgical samples were tested for mRNA expression of IL-1α, IL-1β, IL-18 and TNF-α by real time PCR (**a**), and protein expression in lysates (**b**) or in the supernatants (**c**) detected by ELISA.
