# Supplementary material for: The IL-1/IL-1 receptor axis and tumor cell released inflammasome adaptor ASC are key regulators of TSLP secretion by cancer associated fibroblasts in pancreatic cancer
Source: J Immunother Cancer. 2019 Feb 13;7:45. doi: 10.1186/s40425-019-0521-4 (PMC6373075; doi:10.1186/s40425-019-0521-4)
Supplement: Supplementary file 5 — Figure S5. IL-1β expression in the stroma is mainly supported by TAMs. Immunofluorescence staining was performed to identify IL-1β secreting cells in the tumor stroma. TAM are stained in red (i.e., CD163), IL-1β in green. (DOCX 248 kb) [file 40425_2019_521_MOESM5_ESM.docx]

**Additional File 5: Supplementary Figure S5**


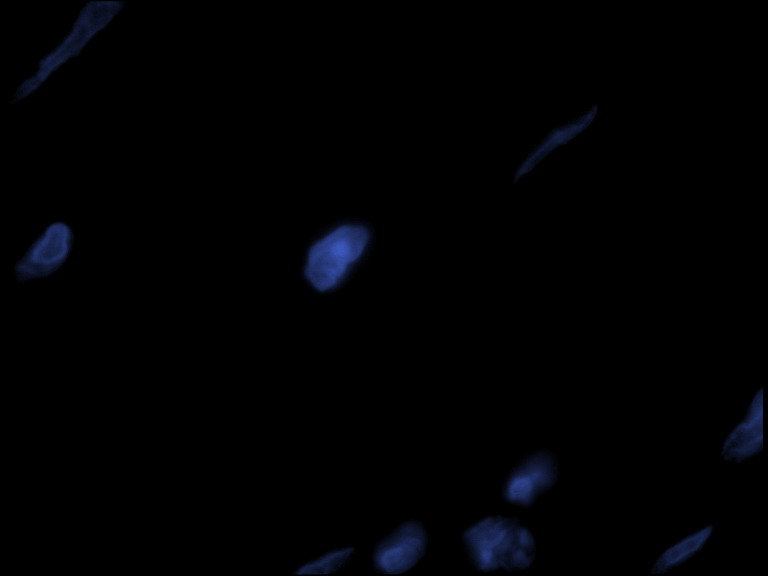

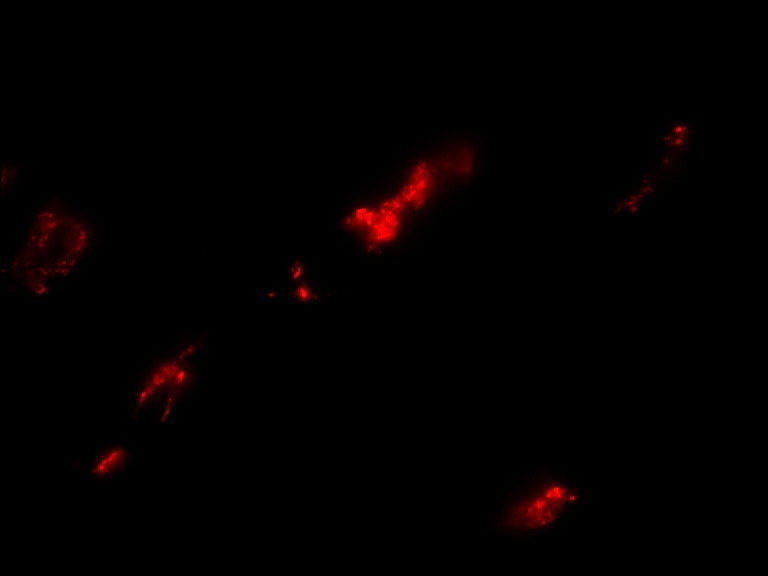

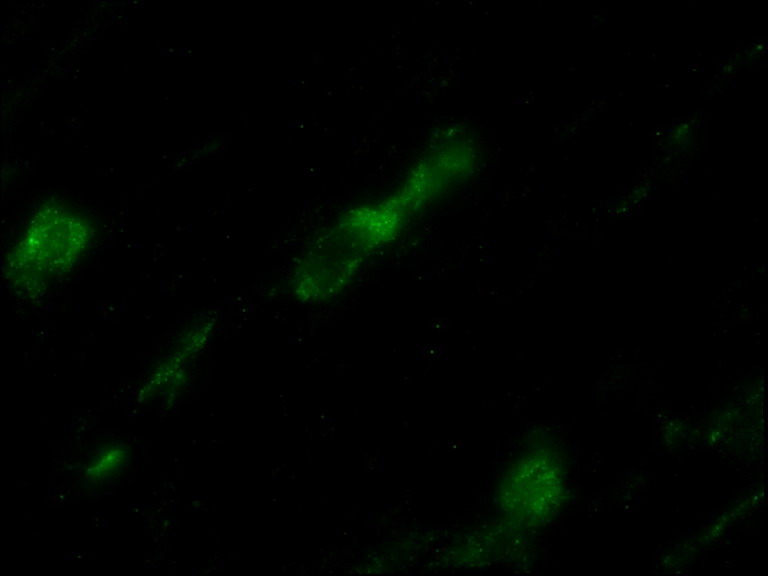

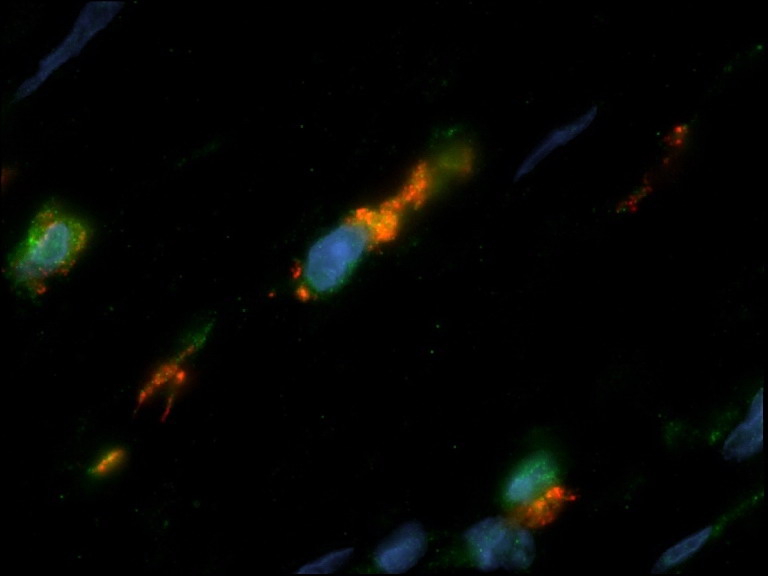


1000x

1000x

1000x

1000x

DAPI

CD163

IL-1β

Merge

**Figure S5.** IL-1β expression in the stroma is mainly supported by TAMs. Immunofluorescence

staining was performed to identify IL-1β secreting cells in the tumor stroma. TAM are stained in

red (i.e., CD163), IL-1β in green.
