# Supplementary material for: The IL-1/IL-1 receptor axis and tumor cell released inflammasome adaptor ASC are key regulators of TSLP secretion by cancer associated fibroblasts in pancreatic cancer
Source: J Immunother Cancer. 2019 Feb 13;7:45. doi: 10.1186/s40425-019-0521-4 (PMC6373075; doi:10.1186/s40425-019-0521-4)
Supplement: Supplementary file 6 — Figure S6. Levels of HMGB1 measured by ELISA in the supernatant of the indicated PDAC cell lines. (DOCX 86 kb) [file 40425_2019_521_MOESM6_ESM.docx]

**Additional File 6: Supplementary Figure S6**

**Figure S6.** Levels of HMGB1 measured by ELISA in the supernatant of the indicated

PDAC cell lines.
