# Supplementary figures and images for: The IL-1/IL-1 receptor axis and tumor cell released inflammasome adaptor ASC are key regulators of TSLP secretion by cancer associated fibroblasts in pancreatic cancer
Source: J Immunother Cancer. 2019 Feb 13;7:45. doi: 10.1186/s40425-019-0521-4 (PMC6373075; doi:10.1186/s40425-019-0521-4)

**Additional File 7: Supplementary Figure S7**


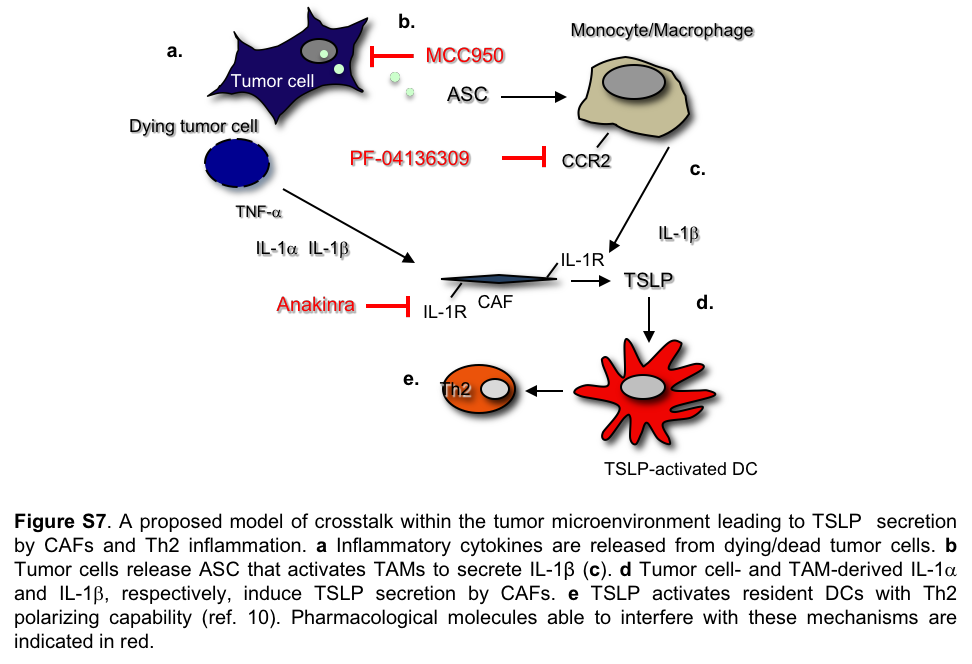

Supplement: Supplementary file 7 — Figure S7. Proposed model of crosstalk within the tumor microenvironment leading to TSLP secretion by CAFs and Th2 inflammation. a Inflammatory cytokines are released from dying/dead tumor cells. b Tumor cells release ASC that activates TAMs to secrete IL-1β (c). d Tumor cell- and TAM-derived IL-1α and IL-1β, respectively, induce TSLP secretion by CAFs. e TSLP activates resident DCs with Th2 polarizing capability. Pharmacological molecules able to interfere with these mechanisms are indicated in red. (DOCX 2538 kb) [file 40425_2019_521_MOESM7_ESM.docx]
